# Isolation, identification, biological characteristics, and antifungal efficacy of sodium bicarbonate combined with natamycin on *Aspergillus niger* from Shengzhou nane (*Prunus salicina* var. taoxingli) fruit

**DOI:** 10.3389/fmicb.2022.1075033

**Published:** 2023-01-12

**Authors:** Tian-Rong Guo, Qing Zeng, Guo Yang, Si-Si Ye, Zi-Yi Chen, Shi-Ying Xie, Hai Wang, Yi-Wei Mo

**Affiliations:** College of Life Science, Shaoxing University, Shaoxing, China

**Keywords:** *Prunus salicina* var. taoxingli, *Aspergillus niger*, sodium bicarbonate, natamycin, antifungal mechanism

## Abstract

The fungi causing fruit rot were isolated from symptomatic Shengzhou nane (*Prunus salicina* var. taoxingli) fruit and were identified as *Aspergillus niger* by biological characteristics and molecular analysis of the internal transcribed spacer region (rDNA-ITS) and translation elongation factor-1α (TEF-1α) sequences. Optimal growth conditions for *A. niger* were 30°C, pH 5.0–6.0, and fructose and peptone as carbon and nitrogen sources. The effects of sodium bicarbonate (SBC), natamycin (NT), and combined treatments on *A. niger* inhibition were investigated. Treatment with 4.0 g/L sodium bicarbonate (SBC) + 5.0 mg/L natamycin (NT) inhibited mycelial growth and spore germination as completely as 12.0 mg/L SBC or 25.0 mg/L NT. SBC and NT treatments disrupted the structural integrity of cell and mitochondria membranes and decreased enzyme activities involved in the tricarboxylic acid (TCA) cycle, mitochondrial membrane potential (MMP), ATP production in mitochondria, and ergosterol content in the plasma membrane, thus leading to the inhibition of *A. niger* growth. Moreover, experimental results *in vivo* showed that the rot lesion diameter and decay rate of Shengzhou nane fruit treated with SBC and NT were significantly reduced compared with the control. The results suggest that the combination treatment of SBC and NT could be an alternative to synthetic fungicides for controlling postharvest Shengzhou nane decay caused by *A. niger*.

## 1. Introduction

Fruit suffers from rapid spoilage as a result of infection with many fungal pathogens, such as *Penicillium digitatum*, *Botritis cinerea*, *Aspergillus*, *Richoderma viridescens*, and *Monilinia fructigena*, that cause decay during storage and marketing and reduce fruit quality (Zhou et al., 2018; Cui et al., 2021; Huang et al., 2021; Xu et al., 2021). Postharvest decay is the most serious disease affecting peach fruit quality and causing a great loss for farmers (Liu et al., 2017; Hashem et al., 2019). Recent investigations have shown that more than one-third of harvested fruit is lost due to pathogen infection. Meanwhile, many fungal pathogens that cause postharvest fruit rot produce toxins that are harmful and potentially carcinogenic to humans (Baggio et al., 2016). Several postharvest synthetic fungicides, including benzoxystrobin, iprodione, prohydantoin, and dysenine, which usually have wide target spectra, are available to control fruit rot (Knight et al., 1997). However, in response to scientific and consumer concerns about human health and environmental safety caused by synthetic fungicides due to possible toxicological risks, as well as the increasing regulatory restrictions on the postharvest use of synthetic chemicals (Dezman et al., 1986), synthetic fungicides are being withdrawn from the market. Therefore, the need for an alternative strategy to control postharvest fruit diseases has been the focus of considerable research.

Shengzhou nane (*Prunus salicina* var. taoxingli), a main cultivated plum with high nutrition and pleasant flavor, is native to Zhejiang, China (Xu et al., 2020). Shengzhou nane usually ripens in mid-July in summer, and the high ambient temperature makes it susceptible to microbial infection during harvest (Mo et al., 2017; Yan et al., 2021). In particular, fungi infections result in significant postharvest losses in the market. To lower disease occurrence in Shengzhou nane fruit and increase farmers’ incomes, the disease occurrence needs to be monitored, and preventative measures should be taken to avoid its spread. However, to the best of our knowledge, till present, there have been no verified reports focusing on pathogen identification and cheap, safe, and eco-friendly alternatives to synthetic fungicides for the control of postharvest decay of Shengzhou nane fruit.

Sodium bicarbonate (SBC) is a general food additive, has less risk of phytotoxicity at the low concentrations at which it is used (1–4%; Karabulut et al., 2001; Palou et al., 2001; Usall et al., 2008), and is generally regarded as safe by the United States Food and Drug Administration (FDA) (Lai et al., 2015). Several previous studies have demonstrated that SBC plays an important role in the inhibition of postharvest diseases in several fruits. For example, SBC controls postharvest diseases caused by *P. digitatum* (Zhu et al., 2012), *Penicillium italicum*, *Geotrichum citri-aurantii* (Hong et al., 2014), and *Monilinia fructigena* (Lyousfi et al., 2022). Nonetheless, similar to other alternative treatments, its application alone shows much lower effectiveness compared to synthetic fungicides. Thus, the antibacterial effect of the combined treatments of SBC with other bactericides has been reported (Ippolito et al., 2005; Casals et al., 2010; Lyousfi et al., 2022). Natamycin (NT), a natural antifungal substance, is a polyene macrolide produced by *Streptomyces* spp. during fermentation (Aparicio et al., 2016). NT is effective against fungal pathogens regardless of their fungicide-resistance phenotypes. Accordingly, NT has been widely used as a preservative in certain foods, such as strawberry, mandarin, and lemon (Haack et al., 2018; Saito et al., 2020; Fernández et al., 2022). However, no study has been performed on SB or NT applications for controlling fungal diseases of Shengzhou nane fruit. Therefore, the objectives of this study were (i) to identify the major pathogen infecting Shengzhou nane using morphological features and rDNA-ITS analysis; (ii) to understand the epidemic properties of the pathogen by determining its biological characteristics, including the optimum culture pH, temperature, and sources of carbon and nitrogen for pathogen growth; and (iii) to evaluate the antifungal efficacy of SBC and NT against pathogens and the possible mechanism. The results will provide novel insights into exploring the potential of SBC and NT in controlling pathogen infection of Shengzhou nane fruit.

## 2. Materials and methods

### 2.1. Isolation and identification of fungal strains

Pathogens were isolated from naturally infected Shengzhou nane fruit obtained from an orchard in Shengzhou city, Zhejiang Province, China, and were immediately transported to the laboratory for experiments. The naturally infected tissues were collected at the junction of the diseased and healthy parts of the fruit when they decayed with typical diseases. The samples were sterilized with 75% alcohol for 1 min, soaked with 1.0% NaClO for 5 min, and rinsed with sterile water 3 times. Subsequently, the tissues were transferred to potato dextrose agar (PDA) medium and cultured at 30°C in the dark until mycelia appeared. Morphological traits, namely, color, shape, growth rate of the colony, and size of the spore, were measured using a microscope, and a total of more than 50 conidia were randomly selected for further length and width measurement.

The nuclear ribosomal internal transcribed spacer region (ITS) gene was amplified using the following universal primers: ITS1-F (CTTGGTCATTTAGAGGAAGTAA) and ITS4 R (TCCTCCGCTTAT TGATATGC) (Hashem et al., 2019). The translation elongation factor-1 (EF-1) gene was amplified with EF1-728F and EF1-986R primers (Carbone and Kohn, 1999). Using tweezers, 0.10 g of mycelium was collected from *A. niger* colonies cultured in PDA for 5 days. Samples were rapidly frozen in liquid nitrogen and homogenized to a powder, and the total genomic DNA was extracted using a DNeasy Kit (Beijing Solarbio Science and Technology Co., Ltd., China) following the manufacturer’s instructions. PCR was performed in a 25 μl volume containing a 1.0 μl DNA template, 1.0 μl of each primer (10 μmol/L), 12.5 μl 2 × PCR Taq Master Mix (Tiangen Biotech, China), and 10.5 μl ddH_2_O. The amplification conditions were performed in a thermal cycle as follows: 95°C for 4 min, followed by 34 cycles of 95°C for 30 s, 56.5°C for 30 s, 72°C for 1 min, and 72°C for 10 min. The PCR products were sequenced by Sangon Biotech, Shanghai, China. The ITS and EF-1 sequences were used for BLAST searches in the GenBank database, and the obtained results were compared with those of similar sequences deposited in the National Center for Biotechnology Information (NCBI)^1^ databank. A phylogenetic tree was constructed using MEGA7.0 software. To fulfill Koch’s postulates, 10 healthy Shengzhou nane fruits were inoculated with a pathogen spore suspension (15 μL, 1 × 10^5^ spores/mL) from isolates collected from rotted fruit after surface sterilization, and sterilized water injection was used as the control. All samples were kept in sealed plastic boxes at 30°C with 90% relative humidity for 4 days.

### 2.2. Pathogenicity test

A pathogenicity test was carried out according to the methods described by Hashem et al. (2019) and Huang et al. (2021). Healthy fruit including Shengzhou nane, yellow peach (*Prunus persica*), flat peach (*Prunus persica* “Compressa”), nectarine (*Prunus persica* var. nectarine), snow pear (*Pyrus* spp.), sand pear (*Pyrus pyrifolia*), apple (*Malus domestica*), carmine plum (*Prunus salicina*), red plum (*Prunus persica*), black plum (*Prunus persica*), cherry (*Prunus avium* L.), jujube (*Ziziphus jujuba* Mill.), tomato (*Solanum lycopersicum*), and grape (*Vitis vinifera* L.), which are the main fruits cultivated in the local area and easily decayed by fungi, were obtained from local orchards without apparent damage or disease on the surface. Six fruits were soaked in 1.0% NaClO for 2 min, washed with sterile water, air dried, and wounded using a sterilized steel rod. Fifteen microliters of the spore suspension (1 × 10^5^ spores/mL) was used to inoculate each healthy fruit (three replicates), while another three healthy fruits were inoculated with sterilized water as the control. All inoculated fruit was kept in sealed plastic boxes at 25°C with 90% relative humidity. The results were observed after 3 days of incubation.

### 2.3. Biological characteristics of the pathogen

To understand its epidemic properties, the biological characteristics of the pathogen, including optimal temperature, pH, carbon (C) source, and nitrogen (N) source for pathogen growth, were evaluated. The isolate was incubated on the PDA medium at 15, 20, 25, 30, 35, and 40°C and at pH 6.0 in the dark for 4 days. Furthermore, four lethal temperatures of 50–75°C were evaluated in a hot water bath for mycelia growth. Meanwhile, the isolate was inoculated on PDA at a pH of 4, 5, 6, 7, 8, 9, 10, and 11 at 30°C in the dark for 4 days. The pH of the medium was adjusted to 1.0 mol/L HCl or NaOH.

To determine the optimal C and N source, according to the formula of Czapek-Dox agar medium (3.0 g/L NaNO_3_, 1.0 g/L KH_2_PO_4_, 0.5 g/L MgSO_4_·7H_2_O, 0.5 g/L KCl, 0.01 g FeSO_4_, 30.0 g/L sucrose, 20 g/L agar, 1,000 mL H_2_O, pH 7.0), 31.58 g/L glucose, 31.93 g/L sorbitol, 31.93 g/L maltose, 31.58 g/L fructose, 28.42 g/L starch, 31.58 g/L galactose or 28.42 g/L xylose instead of 30.0 g/L sucrose as a C source, and 10.58 g/L beef extract, 8.08 g/L peptone, 2.65 g/L glycine, 5.82 g/L phenylalanine, 1.54 g/L arginine, 1.41 g/L N_a_NO_3_, or 1.06 g/L urea instead of 3.0 g/L NaNO_3_ as an N source were used. A control without a C or N source was used for each experiment. The isolate was incubated at pH 6.0 and 30°C in the dark for 4 days. The colony diameter was measured daily in all treatments using the decussation method.

### 2.4. Antifungal efficacy of SBC and NT against mycelia growth

Based on the antifungal activities of SBC and NT (Sigma Aldrich), the effect of SBC and NT on the mycelia growth of the pathogen was measured following protocols described by Zhou et al. (2018) and Xu et al. (2021). In brief, SBC was added to sterilized PDA to generate media with final concentrations of 0.0, 2.0, 4.0, 6.0, 8.0, 10.0, and 12.0 g/L; meanwhile, NT was added to sterilized PDA to generate media with final concentrations of 0.0, 2.5, 5.0, 10.0, 15.0, 20.0, and 25.0 mg/L. In addition, SBC and NT were both added to sterilized PDA to generate media with final concentrations of 6.0 g/L SBC + 2.5 mg/L NT and 6.0 g/L SBC + 5.0 mg/L NT according to the pretest results. The medium was poured into glass Petri dishes, and a 10 μL spore suspension (1 × 10^5^ spores/mL) of the pathogen was used to inoculate the PDA at 4 different points. Then, the Petri dishes were incubated at 30°C for 4 days, and the colony diameter was measured daily using the decussation method. The antifungal effect of the chemistry was evaluated according to Fadda et al. (2021). In brief, the antifungal effect of the plant extract was evaluated when the control colonies completely covered the plate surface. The results were expressed as a percentage of growth inhibition, calculated as follows:


GI=100∗(G–g)/G,

where GI is the growth inhibition, G is the growth (cm) of the control without extract, and g is the growth of the colony in the media with extracts. The experiment was repeated three times.

### 2.5. Antifungal efficacy of SBC and NT against spore germination

**Figure 1:**
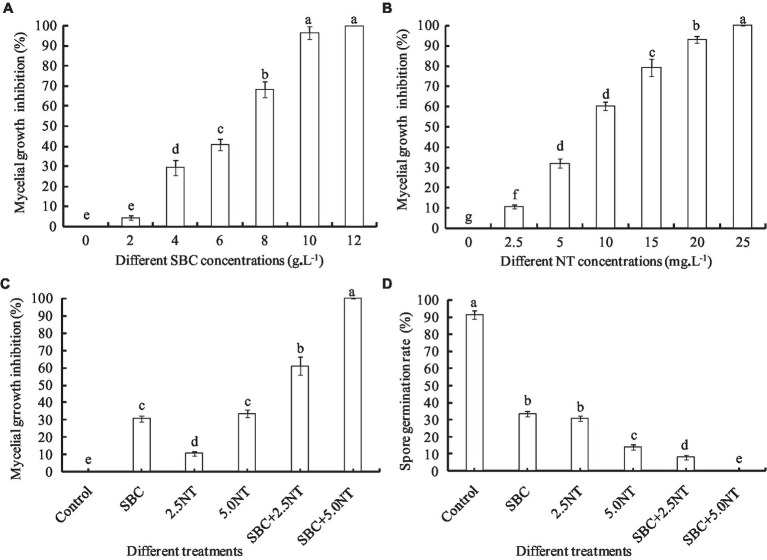
Effect of SBC, NT, and combination treatments on the mycelial growth and spore germination of *Aspergillus niger*. **(A)** Effect of SBC on colony growth. **(B)** Effect of NT on colony growth. **(C)** Effect of SBC and NT combinations on colony growth. **(D)** Effect of SBC and NT combination on spore germination rate. SBC means 6.0 g/L NaHCO_3_; 2.5NT means 2.5 mg/L natamycin; 5.0NT means 5.0 mg/L natamycin; SBC + 2.5NT means 4.0 g/L NaHCO_3_ + 2.5 mg/L natamycin; and SBC + 5.0NT means 4.0 g/L NaHCO_3_ + 5.0 mg/L natamycin. ANOVA was used to compare the mean values of analysis parameters (*p* < 0.05). The results are shown as the mean ± standard deviation (*n* = 3). Different superscript letters in the same column indicate significant differences (*p* < 0.05).

**Figure 2:**
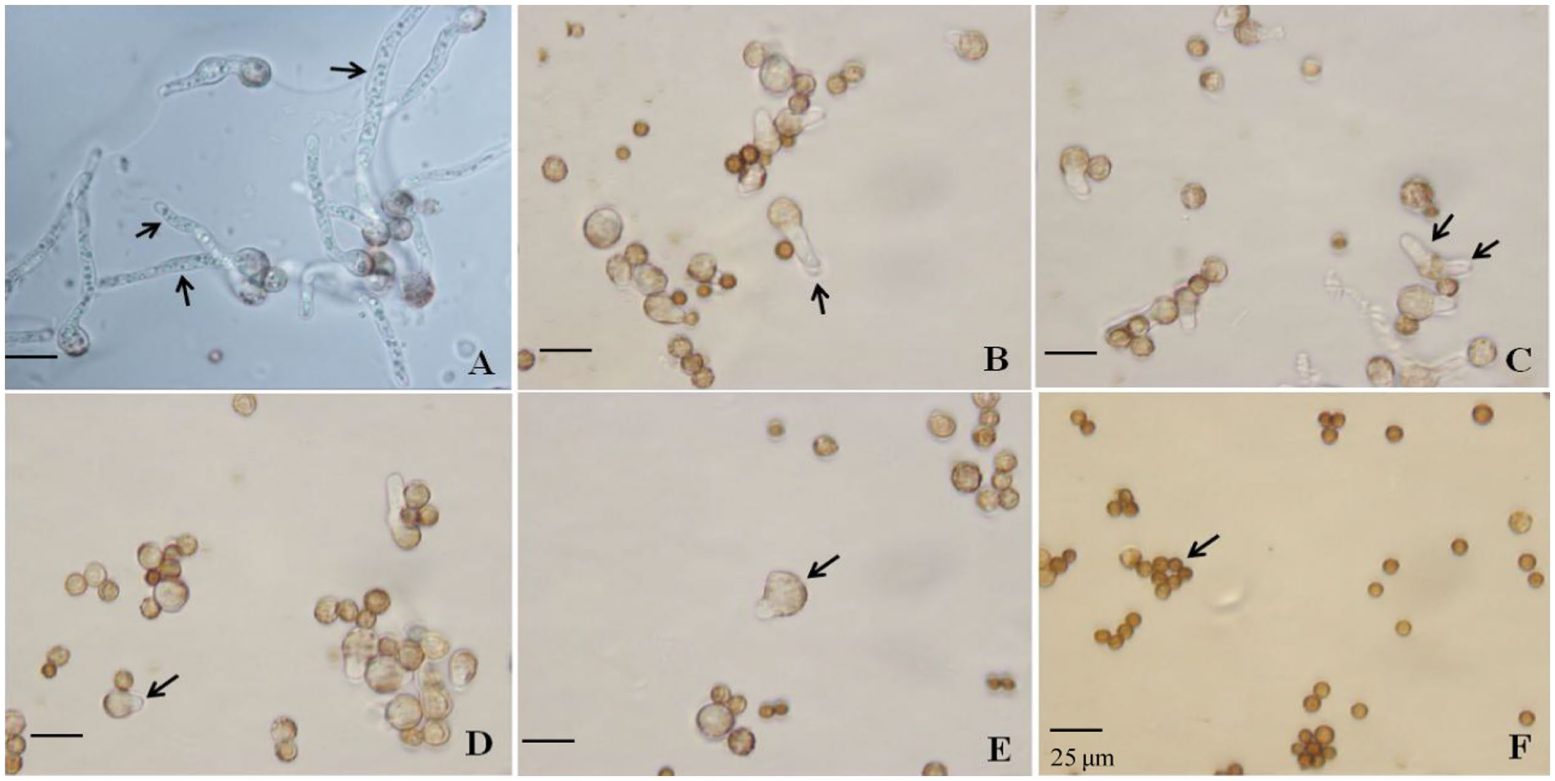
Effect of SBC, NT, and the combination treatment on spore germination of *Aspergillus niger*. **(A)** Control; **(B)** 6.0 g/L SBC; **(C)** 2.5 mg/L NT; **(D)** 5.0 mg/L NT; **(E)** 4.0 g/L SBC + 2.5 mg/L NT; **(F)** 4.0 g/L SBC + 5.0 mg/L NT, 24 h after inoculation. Images were obtained by microscopy at 1,000× magnification, bar = 25 μm.

Spore germination was very sensitive to chemistry culture; thus, several lower concentrations of SBC and NT were applied to evaluate the antifungal efficacy of SBC and NT against spore germination, based on our pretest results. The effects of SBC and NT on the spore germination of *A. niger in vitro* were assessed according to Jiao et al. (2020). Briefly, the solutions of 6.0 g/L SBC, 2.5 mg/L NT, 5.0 mg/L NT, and the combinations of 6.0 g/L SBC + 2.5 mg/L NT and 6.0 g/L SBC + 5.0 mg/L NT were prepared in 20 mL sterile potato dextrose broth (PDB) medium containing an *A. niger* suspension (1 × 10^5^ spores/mL). The samples in cotton-plugged glass tubes were cultivated at 30°C. After incubation for 24 h, the conidia were washed at least two times with phosphate-buffered saline (PBS, pH 7.0) and resuspended with sterile water. Subsequently, 20 μL of the spore suspension was added to a concave slide with a sterile pipette. From each slide, 200 spores were counted at random using an optical microscope. A spore was considered germinated when the length of the germinal tubule reached two times that of the total spore diameter. The inhibition percentage of spore germination was determined with respect to the control. The percentage of germinating spores among all spores detected was determined using a light microscope at a magnification of 1,000×. The average of the data obtained from the three replications is presented (Figures 1, 2).

### 2.6. Plasma membrane integrity of the pathogen

Propidium iodide (PI) staining, following the methods described by Zhou et al. (2018) and Kong et al. (2019) with some modifications, was used to test the membrane integrity of the pathogen mycelia and spores. According to our pretest results, 6.0 g/L or higher SBC, 10.0 mg/L or higher NT, and lower concentrations of the combined SBC and NT treatments destroyed the cell structure. Mycelia and spores treated with 6.0 g/L SBC, 10.0 mg/L NT, and 4.0 g/L SBC + 2.5 mg/L NT were cultured for 4 days. Mycelia were collected from *A. niger* colonies cultured in PDA for 5 days using tweezers. Spores were collected from 5-day-old cultures of fungi growing in a PDA medium. The spores were collected from the plates, rinsed with 5 mL sterile distilled water containing 0.1% Tween-80, filtered through five layers of sterile cheesecloth to remove hyphae, counted by a hemocytometer, and used in the next test. The collected mycelia and spores were incubated with PI at a final concentration of 10.0 μg/mL and kept in the dark at 37°C for 30 min. The mycelia and spores were then collected and washed three times with 50 mmol/L PBS (pH 7.2). Samples were observed with a fluorescence microscope.

Membrane integrity was also assayed using a flow cytometer, following the method described by Wang et al. (2015) and Li et al. (2017), with some modifications. In brief, a 100 μL spore suspension of *A. niger* (1 × 10^5^ spores/mL) was added to 5 mL of 50 mmol/L PBS (pH 7.2) with 2% (w/v) D-glucose containing 0 (control), 6.0 g/L SBC, 10.0 mg/L NT, or 4.0 g/L SBC + 2.5 mg/L NT. They were incubated on a rotary shaker (120 r/min) for 24 h at 30°C. The spore suspension was then centrifuged at 5,000 × *g* for 10 min at 4°C, washed two times with PBS, and filtered two times through a 400 mesh screen to remove cell debris and aggregates. Subsequently, the spores were resuspended in 500 μL of 100 mmol/L PBS (pH 7.2) and stained with PI (10.0 μg/mL) for 30 min at 30°C in the dark. The mixture was centrifuged at 5,000 × *g* for 5 min at 4°C; the precipitate was washed two times with PBS to remove the residual dye, and then resuspended in 500 μL of PBS. Unstained spore suspension was used as an auto-fluorescence control. The spores were detected using a CytoFLEX flow cytometer (BD Biosciences). Fluorescence intensity upon stimulation with an argon-ion laser at 488 nm was measured using the PMT4 channel (625DL filter) and plotted against the cell number. From each sample, 20,000 spores were sorted and analyzed. The percentage of fluorescent spores in the population was calculated. Three replicates of each treatment were performed, and the entire experiment was performed three times.

### 2.7. Mitochondrial activities of the pathogen

*Aspergillus niger* treated with 6.0 g/L SBC, 10.0 mg/L NT, and 4.0 g/L SBC + 2.5 mg/L NT were cultured for 4 days. Collected mycelia and spores were used to determine the mitochondrial activity, the enzyme activities involved in the tricarboxylic acid (TCA) cycle, MMP, ATP, and ergosterol contents, as shown below.

Rhodamine 123 and a mitochondrial red fluorescence probe were used to test the mitochondrial activity. Mycelia and spores were incubated with Rhodamine 123 (50.0 μg/L) for 30 min and the mitochondrial red fluorescence probe (1.0 μg L^−1^) for 1.0 h in the dark at 37°C. Mycelia and spores were collected and washed three times with 50 mmol/L PBS (pH 7.2). Samples with different stained proportions were observed with a fluorescence microscope to evaluate the mitochondrial activity.

### 2.8. Enzyme activities involved in the TCA cycle and mitochondrial membrane potential (MMP)

To prepare mitochondria, 2.0 g of mycelia was added to a mortar with a small amount of liquid nitrogen and ground to break up whole cells. Broken cells were suspended with a five-fold volume of mitochondrial extract buffer. The mitochondrial extract buffer was composed of 50 mM potassium phosphate buffer (pH 8.0), 0.3 M mannitol, 0.5% (w/v) bovine serum albumin (BSA), 0.5% (w/v) polyvinylpyrrolidone-40 (PVP-40), 2 mM EGTA, and 20 mM cysteine. The homogenate was squeezed through a 40 × 40 μm mesh nylon cloth and centrifuged at 2,000 × *g* for 15 min. The supernatant was centrifuged at 12,000 × *g* for 15 min. The precipitate was suspended in wash medium buffer [0.3 M mannitol, 0.1% (w/v) BSA and 10 mM TES (pH 7.5)] and centrifuged again at 12,000 × *g* for 15 min. The final precipitate was washed once with wash medium and suspended in a small volume of the washing medium (mitochondrial fraction). The precipitate was used to determine enzyme activity and MMP.

Citrate synthetase (CS), isocitrate dehydrogenase (IDH), α-ketoglutarate dehydrogenase (α-KGDH), succinate dehydrogenase (SDH), and malate dehydrogenase (MDH) reagent kits were purchased from the Nanjing Jiancheng Bioengineering Institute (Nanjing, China). CS, MDH, SDH, IDH, and α-KGDH activities in the mitochondria of the pathogen were determined spectrophotometrically. The reagent kits were used following the manufacturer’s instructions. MDH, SDH, and IDH activities were estimated at 340 nm in terms of redox reactions. α-KGDH and CS activities were determined at 600 and 412 nm, respectively (Kong et al., 2021).

Mitochondrial membrane potential, as a key indicator of mitochondrial function, can reflect the cellular health status (Li et al., 2020). The MMP Detection Kit (JC-1; Jiancheng Bioengineering Institute, Nanjing, China) was used as a fluorescent probe, as it is a rapid and sensitive kit for assessing changes in MMP in cells. It can be used for the early detection of cell apoptosis. First, a working solution of JC-1 was prepared. A positive control was then set up, and the experiment was carried out. Staining was observed using laser confocal microscopy. The JC-1 monomer (green fluorescence) was determined at an excitation wavelength of 490 nm and an emission wavelength of 530 nm. The JC-1 polymer (red fluorescence) was determined at an excitation wavelength of 525 nm and an emission wavelength of 590 nm.

### 2.9. ATP and ergosterol content in mycelia

ATP extraction from mycelia was carried out according to the methods reported by Mo et al. (2013). Typically, fresh mycelia (1.5 g) were rapidly frozen in liquid nitrogen and homogenized to powder. ATP was extracted from the powder with 6.0 mL of 0.6 mol/L perchloric acid in an ice bath for 5 min. The extraction mixture was centrifuged at 8,000 *g* (Heraeus Multifuge X1R, United States) for 15 min at 4°C. The supernatant was then collected and quickly neutralized to pH 6.5–6.8 with 1.0 mol/L NaOH solution. The neutralized supernatant was placed in an ice bath for 40 min to precipitate most of the potassium perchlorate and subjected to filtration paper to remove potassium perchlorate. The filtrate solution was filtered through a 0.45 μm filter. The samples were analyzed with an LC-20 AD HPLC system (Japan) equipped with an LC-20 AD pump system and an SPD-20 AD diode array detector. The column effluent was monitored by absorbance at 259 nm. Ten microliter samples were applied to an Ultrasphere ODS EC 250 × 4.60 mm column (CTO-10AS VP Japan). The materials were eluted with the mobile phase (pH 6.8, 50.0 mmol/L NaH_2_PO_4_) at 1.0 mL/min. The entire running time was about 20 min for the full separation and determination of ATP. ATP in mycelium samples was identified by comparison with the retention time of the standards. Standard ATP (Sigma, United States) was used for quantitative detection, and its concentration was determined using the external standard method.

Ergosterol was extracted as described by Wang et al. (2018). Mycelia were centrifuged at 8,000 × *g* for 10 min and washed two times with distilled water. Then, mycelia (1.5 g) were rapidly frozen in liquid nitrogen and homogenized to powder. A quantity of 15 ml KOH-C_2_H_5_OH solution (30%, w/w) was added to each pellet and vortexed. The mixture was incubated at 85°C for 4 h. After the tubes were cooled to room temperature, 10 mL of ether was added and vortexed. The ergosterol of the upper phase was sampled and quantified using the LC-20 AD HPLC system (Japan). Sterol extracts were separated on a C-18 packed column (245 × 4.6 mm) with methanol at a flow rate of 1 mL/min, and the eluate was continuously monitored using a UV spectrophotometer at 280 nm. Standard ergosterol (Sigma, United States) was used for quantitative detection. The ergosterol content was quantified as milligrams of ergosterol per gram of dry weight (mg/g).

### 2.10. SBC and NT against Shengzhou nane fruit rot *in vivo*

The effects of SBC and NT on controlling postharvest disease *in vivo* were determined following the method described by He et al. (2019). Shengzhou nane was wounded with a sterile nail at the equator before inoculation. Then, the fruit was inoculated with a 10 μl spore suspension (1 × 10^5^ spores/mL) on each wound. After the fruit was air dried for 3 h, 10 μl SBC_1_ (2.0 g/L SBC), SBC_2_ (4.0 g/L SBC), SBC_3_ (6.0 g/L SBC), NT_1_ (25.0 mg/L NT), NT_2_ (50.0 mg/L NT), NT_3_ (100.0 mg/L NT), NT_4_ (200.0 mg/L NT), NT_5_ (400.0 mg/L NT), (SBC + NT)_1_ (4.0 g/L SBC + 25.0 mg/L NT), (SBC + NT)_2_ (4.0 g/L SBC + 50.0 mg/L NT), (SBC + NT)_3_ (4.0 g/L SBC + 100.0 mg/L NT), or (SBC + NT)_4_ (4.0 g/L SBC + 150.0 mg/L NT) were added to the wound sites. In addition, water-treated fruit was used as the control. Treated fruit was placed in a plastic box, and each tray was enclosed with a polyethylene bag to maintain high humidity (95%). The lesion diameter and decay rate of the fruit were used to determine disease severity 4 and 6 days after treatment. Each treatment contained three replicates, with 18 fruits per replicate.

### 2.11. Statistical analysis

All experiments were carried out with at least three replicates. Data were analyzed using one-way ANOVA in SPSS 23 software (SPSS Inc., Chicago, IL, United States). Statistical significance was followed by Tukey’s HSD test at *p* < 0.05 to examine differences between treatments.

## 3. Results

### 3.1. Isolation and identification of fungal strains

**Figure 3:**
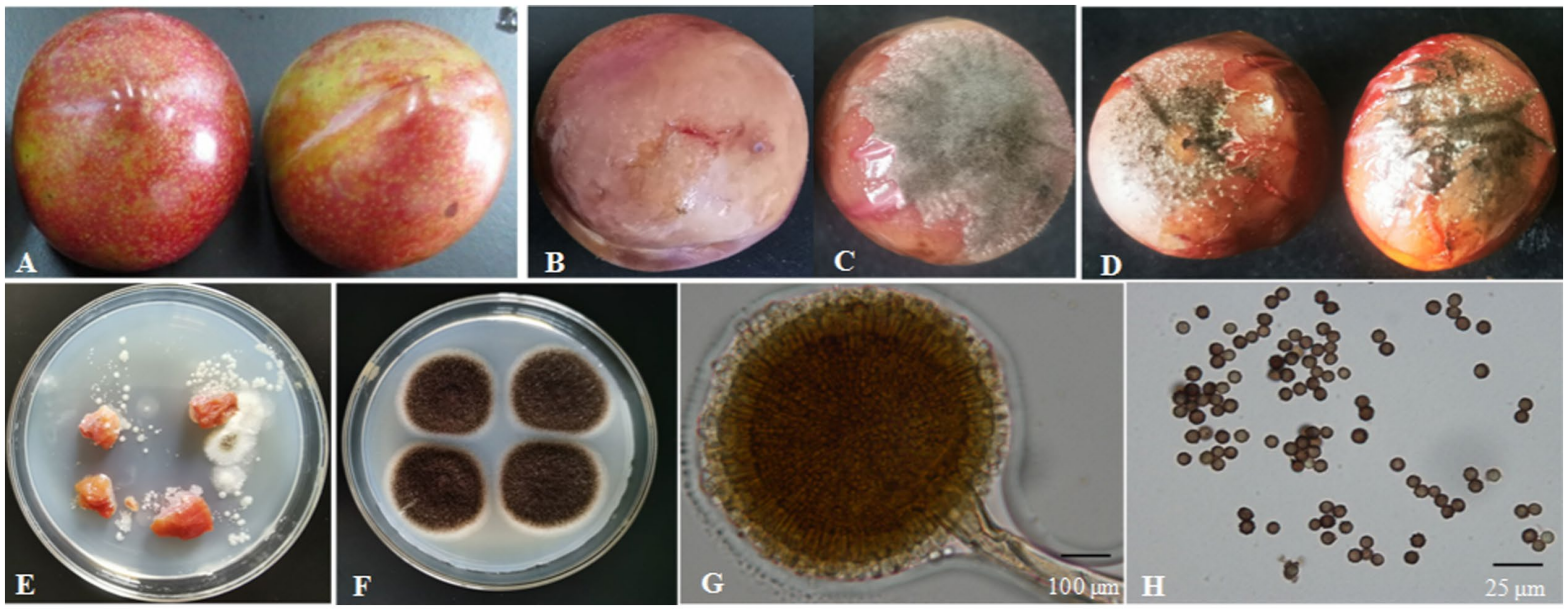
Isolation of the fungal strain from naturally infected fruit. **(A)** Healthy fruit; **(B,C)** naturally infected fruit; **(D)** fruit 4 days after inoculation with fungi for 4 days; **(E)** fungi isolated from naturally infected fruit; **(F)** fungi colony cultured for 4 days on PDA; **(G)** conidium and sporangium, 200×, bar = 100 μm; **(H)** spore, 1,000×, bar = 25 μm.

The surface of the healthy Shengzhou nane fruit was smooth, with bright skin (Figure 3A). However, the peel became pale, and the fruit started to go rotten after being infected by the pathogen; then, black mold appeared (Figures 3B, C). The pathogen from a typical decay Shengzhou nane fruit was isolated and purified by growth on PDA (Figure 3E). After culturing at 30°C in the dark for 4 days, round and aerial hyphae were observed from the colony appearance (Figure 3F). Furthermore, the colony was white in the initial stage, then became dark and extended outward in a strip with no marginal or adherent growth (Figure 3F). Colonies grew with a mean hyphae growth rate of 9.42 ± 0.28 mm per day on PDA in the dark at 30°C. Mycelia were characterized by transverse septa and branches. The spore shape was round, and the size was 3.27 ± 0.13 μm (*n* = 50). The outside surface was uneven due to its sporulation with a single bottle-stem type, while many irregular protrusions were observed on the spore surface (Figures 3G, H).

**Figure 4:**
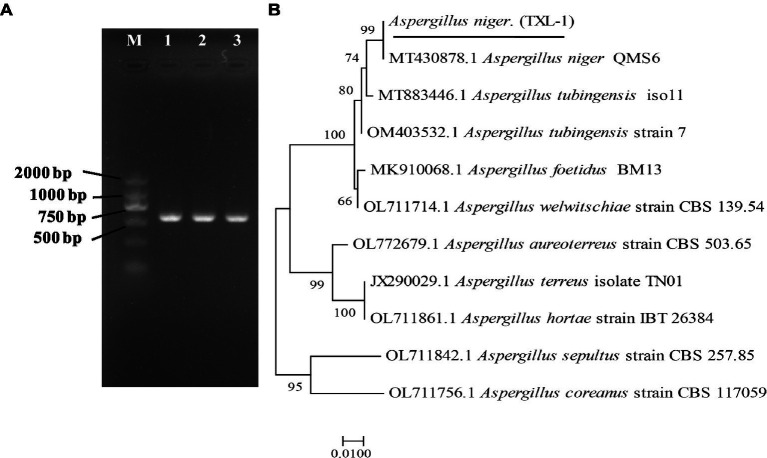
Molecular identification of fungal strains. **(A)** PCR results of the pathogen using DNA-ITS; **(B)** phylogenetic tree based on ITS sequences; (M) marker DL2000; (1, 2, 3) three replications.

A DNA-ITS sequence of about 681 bp was extracted from the isolated DNA (Figure 4A). The ITS regions of the fungal genes were sequenced and aligned to entries in the NCBI sequence database. The Blastn results indicated that ITS sequences shared 99% similarity with *A. niger*. Phylogenetic analysis was conducted using MEGA7.0 software, and the results showed that the isolated colony was clustered with the MT 430878.1 *A. niger* clade (Figure 4B).

**Figure 5:**
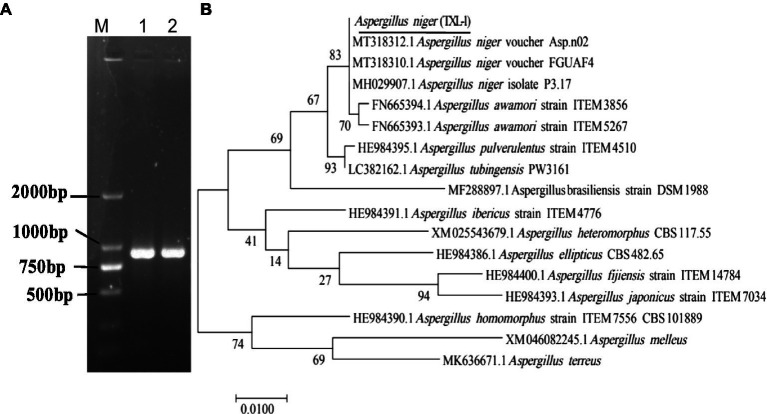
Molecular identification of fungal strains. **(A)** PCR results of the pathogen using EF-1 sequences; **(B)** phylogenetic tree based on EF-1 sequences; (M) marker DL2000; (1, 2) two replications.

The translation elongation factor-1 (EF-1) gene sequence was also amplified with EF1-728F and EF1-986R primer pairs (Figure 5A). The representative sequence was submitted to GenBank. The Blastn results indicated that the EF-1 sequence shared 100% similarity with a strain of *A. niger* (Accession Nos. MT318312.1 and MT318310.1). The isolated colony was clustered with the *A. niger* clade (Figure 5B). Therefore, the isolate was identified as *A. niger* (TXL-1).

In Koch’s postulate experiment, 10 healthy Shengzhou nane fruits were inoculated with isolated *A. niger*. The inoculated fruit became infected, and the same symptoms were found in naturally infected fruits, while those in the control group remained symptomless after 4 days of culture (Figure 3D).

### 3.2. Pathogenicity tests

**Figure 6:**
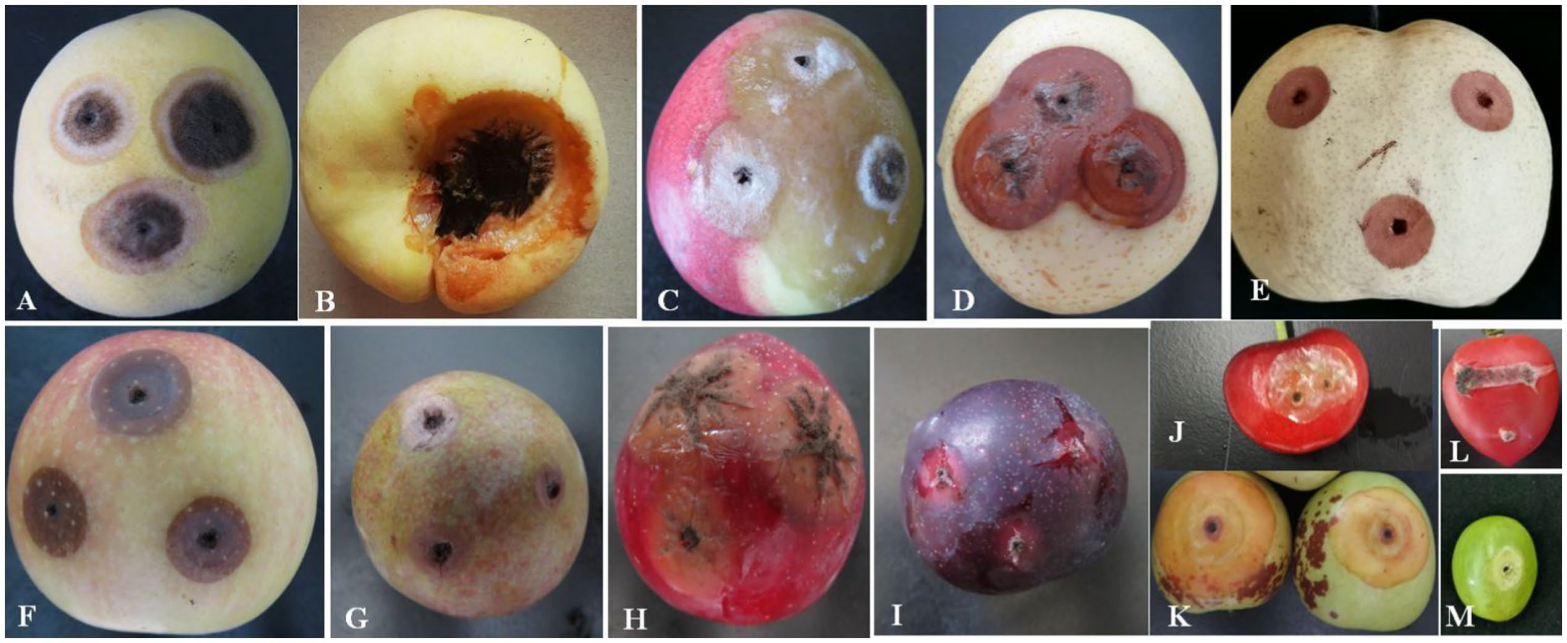
Pathogenicity of *A. niger* to different local fruits. **(A)** Yellow peach; **(B)** flat peach; **(C)** nectarine; **(D)** snow pear; **(E)** sand pear; **(F)** apple; **(G)** carmine plum; **(H)** red plum; **(I)** black plum; **(J)** cherry; **(K)** jujube; **(L)** tomato; and **(M)** grape.

The pathogenicity test of *A. niger* was determined on the fruits of yellow peach, flat peach, nectarine, snow pear, sand pear, apple, carmine plum, red plum, black plum, cherry, jujube, tomato, and grape. The typical round lesions appeared on all fruit surfaces 3 days after inoculation with *A. niger*, which was consistent with the symptoms in those of naturally infected Shengzhou nane fruit. The pathogen was re-isolated from symptomatic rotten fruit. As a result, it was identified as *A. niger* based on its morphological characteristics and gene sequences. Moreover, the pathogenicity of *A. niger* varied in different fruits. For example, mycelium was observed in peach and tomato, but the decay in grapes was not serious (Figure 6).

### 3.3. Biological characteristics of *Aspergillus niger*

**Figure 7:**
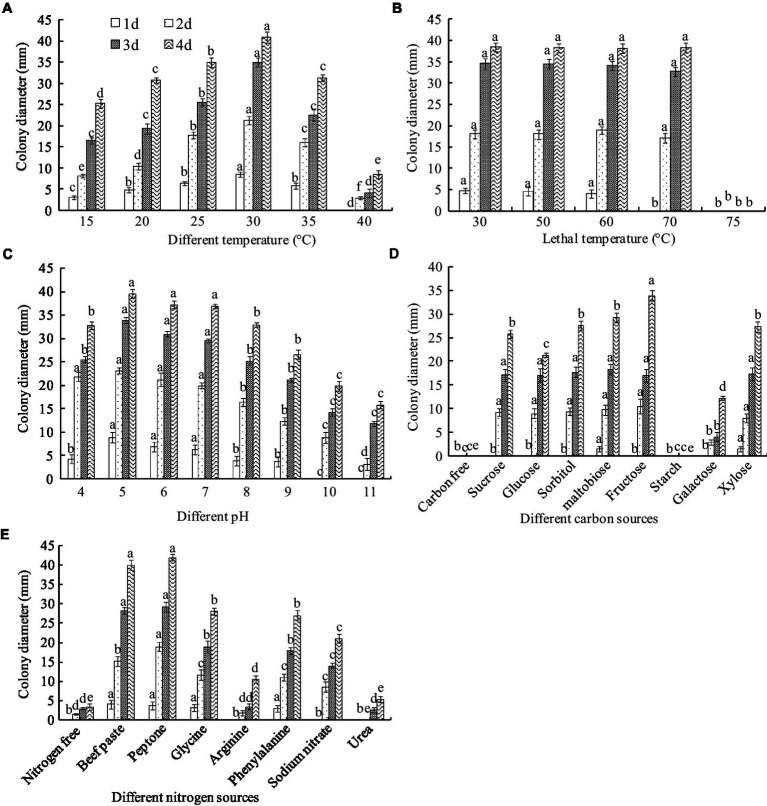
Biological characteristics of *A. niger*. **(A)** Temperature; **(B)** lethal temperature; **(C)** pH; **(D)** carbon sources; **(E)** nitrogen sources. ANOVA was employed to compare the mean values of analysis parameters (*p* < 0.05). The results are shown as the mean ± standard deviation (*n* = 5). Different superscript letters in the same column indicate significant differences (*p* < 0.05).

The biochemical tests showed that *A. niger* grew in a temperature range of 15–40°C. The greatest colony diameter was observed at 30°C (40.83 mm), followed by 25°C (32.93 mm) after 4 days of culture. However, mycelial growth was significantly inhibited at 40°C (Figure 7A). Thus, the optimal culture temperature for *A. niger* growth was 30°C.

To clarify the endurance temperature, the spores were treated with a hot water bath from 50 to 75°C for 10 min. Complete inactivation of the spore occurred at 75°C. However, there was no significant difference in colony diameter between treatments below 75°C (Figure 7B). These results suggest that the lethal temperature for *A. niger* is 75°C for 10 min.

*Aspergillus niger* grew in a pH range of 4.0–11.0. The greatest colony diameter was observed at pH 5.0 and 6.0 but decreased at pH values higher than 8.0 (Figure 7C).

Fructose as a C source in the medium produced the greatest colony diameter, recorded as 33.88 mm after 4 days of culture, followed by sucrose, maltobiose, xylose, and glucose. However, a very thin mycelial density and lower diameter were observed in the galactose media (Figure 7D). Mycelia did not grow in a starch- or carbon-free medium. The results indicated that the optimal C source for *A. niger* growth was fructose.

Peptone and beef paste as N sources in the medium produced the largest colony diameters, recorded as 41.94 and 40.98 mm, respectively, after 4 days of culture, followed by glycine, phenylalanine, sodium nitrate, and arginine. The smallest colony diameters were observed in the urea medium and the control (Figure 7E). The optimal N sources for *A. niger* growth were peptone and beef paste. Interestingly, unlike the C-free medium, the colony grew slowly in the N-free culture, indicating that some other substances could replace the N sources needed for *A. niger* growth.

### 3.4. Antifungal efficacy of SBC and NT against *Aspergillus niger*

Sodium bicarbonate or natamycin caused a significant decrease in the mycelial growth rate in proportion to the concentration (Figures 1A, B). Compared to the control, 6.0 g/L SBC induced mycelial growth inhibition by 40.73% (*p* < 0.05) after inoculation for 4 days, while it increased to 96.48 and 100% at 10.0 and 12.0 g/L SBC, respectively. Mycelial growth was significantly inhibited by all concentrations of NT tested in this experiment, and 25.0 mg/L NT inhibited mycelial growth by 100.0%. Moreover, the combination of SBC and NT induced a higher inhibition efficacy compared with the single chemistry treatments. For example, 4.0 g/L SBC, 2.5 mg/L NT, and 5.0 mg/L NT induced mycelial growth inhibition by 30.55, 10.56, and 33.58%, respectively, whereas the mycelial growth inhibition was 60.93 and 100% under treatments of 4.0 g/L SBC + 2.5 mg/L NT and 4.0 g/L SBC + 5.0 mg/L NT, respectively (Figure 1C).

Spore germination is the key process required to initiate vegetative growth. The germination rate of the spores, as well as the germination time of *A. niger*, differed by treatment. After 24 h incubation in control conditions, 91.28% of spores germinated, whereas the germination rates decreased and were recorded as 35.55, 30.72, 14.23, 5.22, and 0.0% in 6.0 g/L SBC, 2.5 mg/L NT, 5.0 mg/L NT, 4.0 g/L SBC + 2.5 mg/L NT, and 4.0 g/L SBC + 5.0 mg/L NT treatments, respectively (Figure 1D). The spores swelled and were enlarged, and the mycelia grew quickly with many granular inclusions formed in the mycelium after 24 h of culture in the control group during germination. However, only a few spores swelled, but no germination was observed in the SBC or NT treatments. Therefore, spore swelling may be the premise of spore germination. In addition, the 4.0 g/L SBC + 5.0 mg/L NT treatment completely inhibited spore germination (Figure 2).

### 3.5. SBC and NT destroyed the plasma membrane integrity of *Aspergillus niger*

**Figure 8:**
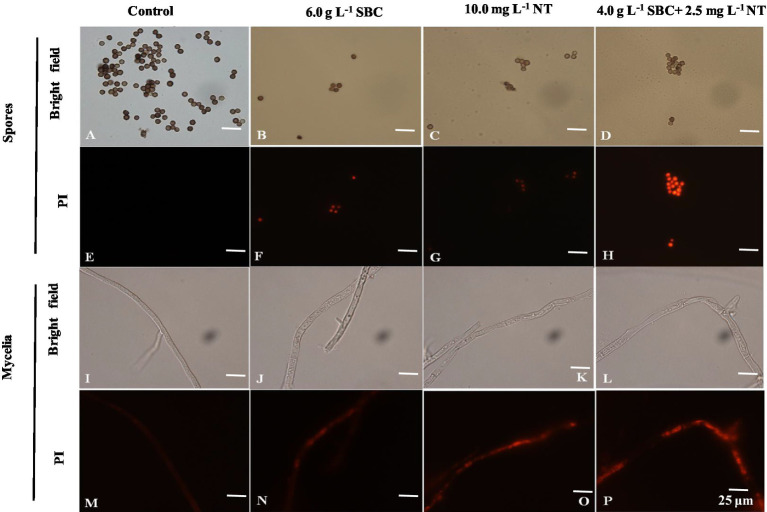
Effect of SBC, NT, and combination treatment on the plasma membrane integrity of *Aspergillus niger*. **(A,E)** Spores in control; **(B,F)** spores treated with 6.0 g/L SBC; **(C,G)** spores treated with 10.0 mg/L NT; **(D,H)** spores treated with 4.0 g/L SBC + 2.5 mg/L NT; **(I,M)** mycelia in control; **(J,N)** mycelia treated with 6.0 g/L SBC; **(K,O)** mycelia treated with 10.0 mg/L NT; **(L,P)** mycelia treated with 4.0 g/L SBC + 2.5 mg/L NT mycelia. Images were obtained by microscopy at 1,000× magnification, bar = 25 μm.

Propidium iodide (PI), a fluorescent molecule, is membrane impermeable and can bind to DNA by intercalating between the bases with less or no sequence preference, which causes the nucleus to be stained with red fluorescence. Therefore, cells stained by PI can be used to identify the extent of cell damage (Jiao et al., 2020). In this study, the PI staining results showed that the mycelia and spores treated with 6.0 g/L SBC, 10.0 mg/L NT, or 4.0 g/L SBC + 2.5 mg/L NT were stained deeply compared to the control (Figure 8), indicating that SBC and NT destroyed the cell membrane integrity of *A. niger* cells and induced mycelia and spore death.

**Figure 9:**
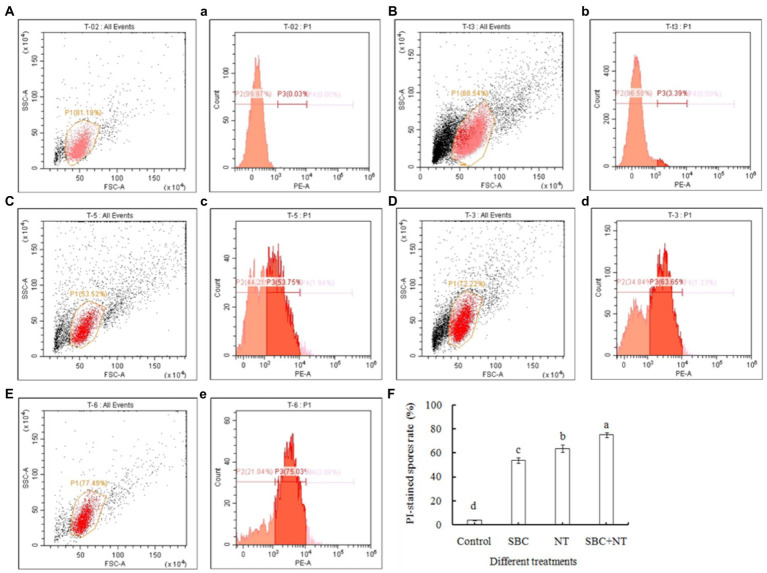
Flow cytometry analysis of the plasma membrane integrity of *Aspergillus niger* spores treated with SBC or NT. **(A–E)** Scattergram showing cell complexity (side scatter, SSC) vs. cell size (forward scatter, FSC); **(a–e)** the corresponding percentage of PI-stained spores compared with the control. **(A,a)** Autofluorescence of non-treated cells; **(B,b)** fluorescence of non-treated cells for PI staining; **(C–E)** cells treated with the varying treatments of SBC **(c)**, NT **(d)**, and SBC + NT **(e)**; **(F)** the percentage of PI-stained spores rate after different treatments. ANOVA was employed to compare the mean values of analysis parameters (*p* < 0.05). The results are shown as the mean ± standard deviation (*n* = 3). Different superscript letters in the same column indicate significant differences (*p* < 0.05).

The membrane integrity of *A. niger* was also determined using flow cytometry after being stained with PI and was presented in two-dimensional dot plots. As shown in Figure 9, a homogenous population of undamaged cells was dominant in the control, while various cell sizes (forward scatter, FSC) and variations in complexity (side scatter, SSC) were observed on a scattergram of *A. niger* spores after 24 h of incubation in SBC and NT. Furthermore, a histogram plot was obtained by counting PI-labeled spores. In the control, the PI-stained spores accounted for only 3.39% of total spores (Figures 9B, b). In treatments of 6.0 g/L SBC (Figures 9C, c), 10.0 mg/L NT (Figures 9D, d), and 4.0 g/L SBC + 2.5 mg/L NT (Figures 9E, e), the percentage of PI-stained spores increased to 53.75, 63.65, and 75.03% (Figure 9F) (*p* < 0.05), respectively, indicating a loss in integrity and heavy damage to the cell membrane of the pathogen.

### 3.6. SBC and NT damage the mitochondrial activity of *Aspergillus niger*

**Figure 10:**
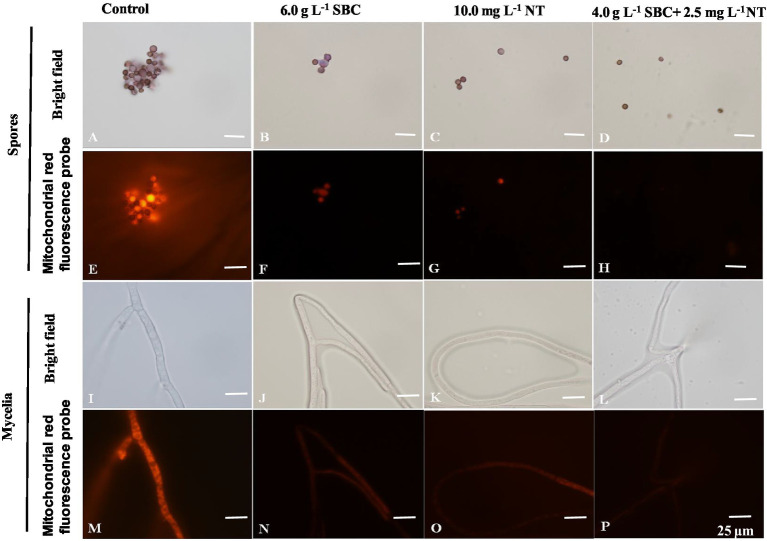
*Aspergillus niger* stained with the mitochondrial red fluorescence probe after treatment with SBC or NT. **(A,E)** Spores in the control; **(B,F)** spores treated with 6.0 g/L SBC; **(C,G)** spores treated with 10.0 mg/L NT; **(D,H)** spores treated with 4.0 g/L SBC + 2.5 mg/L NT; **(I,M)** mycelia in control; **(J,N)** mycelia treated with 6.0 g/L SBC; **(K,O)** mycelia treated with 10.0 mg/L NT; **(L,P)** mycelia treated with 4.0 g/L SBC + 2.5 mg/L NT mycelia. Images were obtained by microscopy at 1,000× magnification, bar = 25 μm.

The mitochondrial red fluorescence probe bound the active mitochondria and emitted red fluorescence at different intensities, according to the MMP of living cells. The spores and mycelia of *A. niger* treated with 6.0 g/L SBC, 10.0 mg/L NT, and 4.0 g/L SBC + 2.5 mg/L NT showed a less stained proportion by mitochondrial red fluorescence compared with the control group (Figure 10).

**Figure 11:**
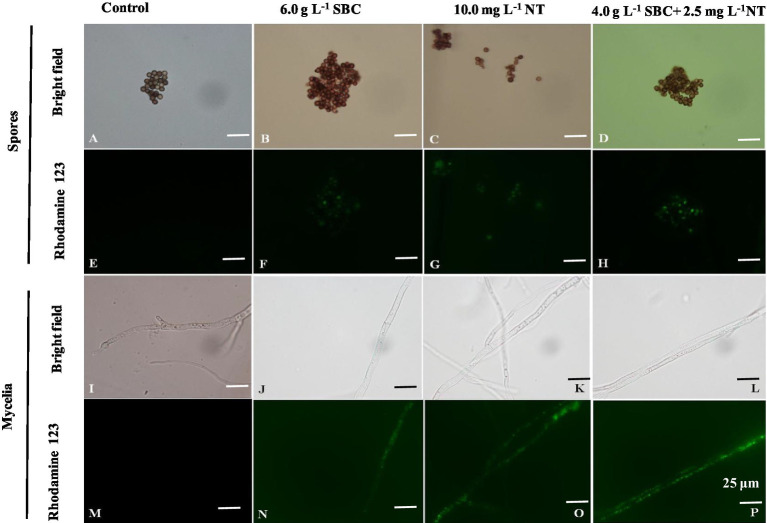
*Aspergillus niger* stained with Rhodamine 123 after treatment with SBC or NT. **(A,E)** Spores in the control; **(B,F)** spores treated with 6.0 g/L SBC; **(C,G)** spores treated with 10.0 mg/L NT; **(D,H)** spores treated with 4.0 g/L SBC + 2.5 mg/L NT; **(I,M)** mycelia in control; **(J,N)** mycelia treated with 6.0 g/L SBC; **(K,O)** mycelia treated with 10.0 mg/L NT; **(L,P)** mycelia treated with 4.0 g/L SBC + 2.5 mg/L NT mycelia. Images were obtained by microscopy at 1,000× magnification, bar = 25 μm.

Rhodamine 123 (Rh-123) is a specific probe used for evaluating changes in MMP since it can accumulate in normal mitochondria and be released to the outside of the mitochondria with a loss of MMP, leading to a stronger green fluorescence signal (Su et al., 2014). As shown in Figure 11, the fluorescence intensity of Rh-123 in the groups treated with 6.0 g/L SBC, 10.0 mg/L NT, and 4.0 g/L SBC + 2.5 mg/L NT was higher than that of the control group. Combined with the fluorescence staining results, it revealed that suitable concentrations of SBC and NT could destroy the cell structure, leading to the hyperpolarization of MMP and causing mitochondrial dysfunction in *A. niger* cells.

### 3.7. SBC and NT decreased the activity of enzymes involved in the TCA cycle

**Figure 12:**
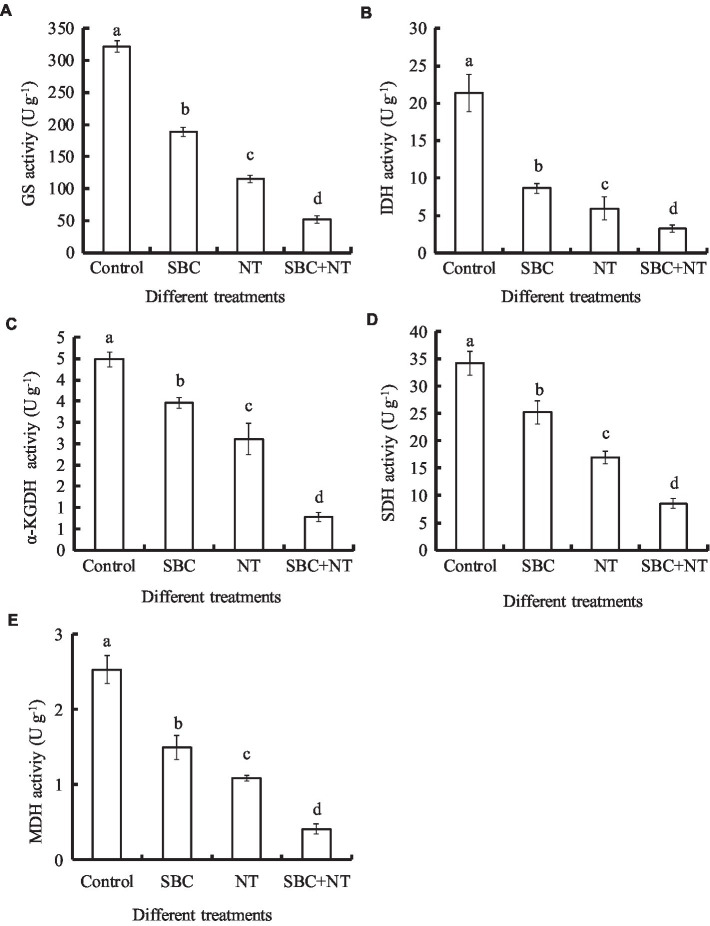
Effect of SBC, NT, and combined SBC and NT on enzyme activities involved in the TCA cycle. **(A)** GS activity; **(B)** IDH activity; **(C)** a-KGDH activity; **(D)** SDH activity; **(E)** MDH activity. ANOVA was employed to compare the mean values of analysis parameters (*p* < 0.05). The results are shown as the mean ± standard deviation (*n* = 3). Different superscript letters in the same column indicate significant differences (*p* < 0.05).

Compared with the control, the activity of the key enzymes involved in the TCA cycle, such as CS, IDH, α-KGDH, SDH, and MDH, in the mitochondria of *A. niger* cells decreased significantly (*p* < 0.05) under the treatments of 6.0 g/L SBC, 10.0 mg/L NT, and 4.0 g/L SBC + 2.5 mg/L NT (Figures 12A–E), especially α-KGDH activity. The significant decreases in the activities of these key enzymes indicated that mitochondrial function was destroyed by SBC and NT treatments.

### 3.8. SBC and NT decreased the ATP content and MMP

**Figure 13:**
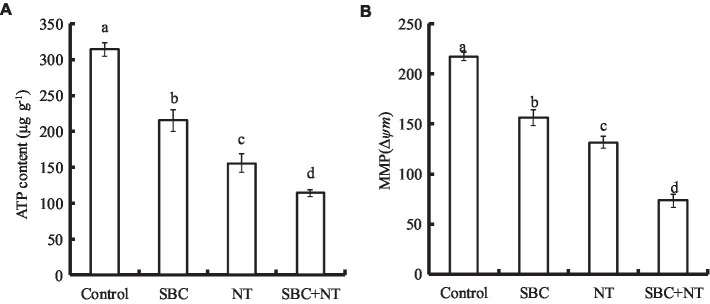
Effect of SBC or NT on ATP content **(A)** and MMP **(B)**. ANOVA was employed to compare the mean values of the analysis parameters (*p* < 0.05). The results are shown as the mean ± standard deviation (*n* = 3). Different superscript letters in the same column indicate significant differences (*p* < 0.05).

The effects of the SBC and NT treatments on the ATP content of *A. niger* are presented in Figure 13A. Compared with the control, 6.0 g/L SBC, 10.0 mg/L NT, and 4.0 g/L SBC + 2.5 mg/L NT decreased the ATP content by 31.50, 50.51, and 63.68%, respectively (*p* < 0.05). Meanwhile, MMP decreased significantly in *A. niger* cells under the treatments of 6.0 g/L SBC, 10.0 mg/L NT, and 4.0 g/L SBC + 2.5 mg/L NT and was recorded as 30.03, 41.51, and 59.51% of the control, respectively (*p* < 0.05) (Figure 13B).

### 3.9. SBC and NT decreased the ergosterol content in mycelia

**Figure 14:**
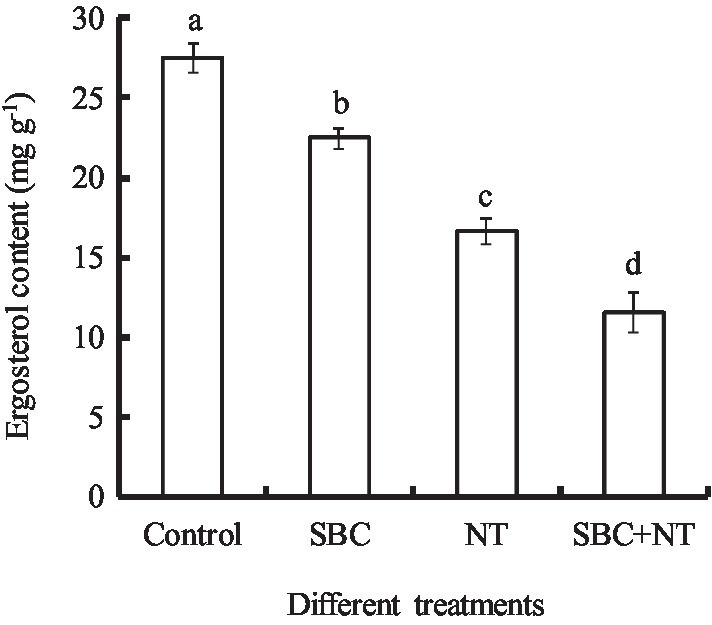
Effect of SBC or NT on ergosterol content in mycelia. ANOVA was used to compare the mean values of analysis parameters (*p* < 0.05). The results are shown as the mean ± standard deviation (*n* = 3). Different superscript letters in the same column indicate significant differences (*p* < 0.05).

The effects of SBC and NT on the ergosterol content in the plasma membranes of *A. niger* mycelia are shown in Figure 14. After incubating with SBC or NT, the total ergosterol content decreased significantly. In detail, the ergosterol content decreased by 18.17, 39.42, and 58.09% (*p* < 0.05) of the control under the treatments of 6.0 g/L SBC, 10.0 mg/L NT, and 4.0 g/L SBC + 2.5 mg/L NT, respectively.

### 3.10. SBC and NT against Shengzhou nane fruit rot caused by *Aspergillus niger*

**Figure 15:**
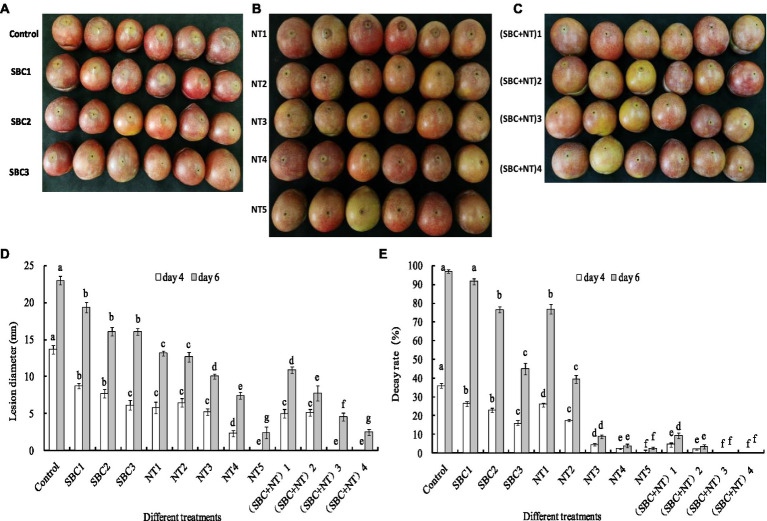
SBC or NT inhibits *Aspergillus niger* virulence on harvested Shengzhou nane fruit. **(A)** Control and SBC treatments; **(B)** NT treatments; **(C)** SBC and NT combined treatments; **(D)** corresponding histograms for statistical data on lesions on harvested fruit; **(E)** fruit decay rate. SBC_1_: 2.0 g/L, SBC_2_: 4.0 g/L, SBC_3_: 6.0 g/L, NT_1_: 25.0 mg/L, NT_2_: 50.0 mg/L, NT_3_: 100.0 mg/L, NT_4_: 200.0 mg/L, NT_5_: 400.0 mg/L, (SBC + NT)_1_: 4.0 g/L SBC + 25.0 mg/L NT, (SBC + NT)_2_: 4.0 g/L SBC + 50.0 mg/L NT, (SBC + NT)_3_: 4.0 g/L SBC + 100.0 mg/L NT, (SBC + NT)_4_: (4.0 g/L SBC + 150.0 mg/L NT). ANOVA was employed to compare the mean values of the analysis parameters (*p* < 0.05). The results are shown as the mean ± standard deviation (*n* = 3). Different superscript letters in the same column indicate significant differences (*p* < 0.05).

Rot lesions were significantly alleviated in Shenzhou nane fruit treated with different concentrations of SBC and NT 4 days after inoculation with *A. niger* spores (Figures 15A–C). The rot lesion diameter in the control fruit reached 13.67 mm. In contrast, it was reduced by 29.33, 43.76, 57.57, 34.79, 52.69, 61.96, 83.02, 100.00, 63.42, 68.30, 100.00, and 100.00% of the control (*p* < 0.05) in fruit treated with SBC_1_ (2.0 g/L SBC), SBC_2_ (4.0 g/L SBC), SBC_3_ (6.0 g/L SBC), NT_1_ (25.0 mg/L NT), NT_2_ (50.0 mg/L NT), NT_3_ (100.0 mg/L NT), NT_4_ (200.0 mg/L NT), NT_5_ (400.0 mg/L NT), (SBC + NT)_1_ (4.0 g/L SBC + 25.0 mg/L NT), (SBC + NT)_2_ (4.0 g/L SBC + 50.0 mg/L NT), (SBC + NT)_3_ (4.0 g/L SBC + 100.0 mg/L NT), or (SBC + NT)_4_ (4.0 g/L SBC + 150.0 mg/L NT), respectively (Figure 15D). Disease occurrence was completely restricted by the treatments NT_5_, (SBC + NT)_3_, and (SBC + NT)_4_ (Figures 15B, C). Six days after inoculation, the fruit became soft, and the rot lesion diameters increased in all treatments. Moreover, the difference in the rot lesion diameters between the control and treatments SBC, NT, and SBC + NT was more marked. For instance, the rot lesion diameter was only 9.17% of the control in the fruit treated with (SBC + NT)_4_. Meanwhile, after inoculation with *A. niger* spores, the decay rate of the fruit treated with NT_5_ and SBC + NT was significantly reduced during storage compared with the control. The decay rate of the control group was 97.0%, while it was 2.66, 9.33, 3.67, 0.0, and 0.0% in the fruit treated with NT_5_, (SBC + NT)_1_, (SBC + NT)_2_, (SBC + NT)_3_, and (SBC + NT)_4_, respectively, on the 6th day of storage (Figure 15E). The results indicated that SBC + NT was effective against *A. niger* in Shengzhou nane fruit.

## 4. Discussion

In this study, *A. niger* isolated from the naturally infected Shengzhou nane fruit was identified by morphological features, rDNA-ITS analysis, and EF-1 analysis. The biochemical tests indicated that the optimal incubation temperature for pathogen growth was 30°C, which was consistent with the temperature at the time of disease outbreak since the daily mean temperature was about 30°C in the Shenzhou nane mature period in the local orchard. However, temperatures below 15°C or above 40°C significantly inhibited mycelial growth. The lethal temperature showed complete inactivation of the spores at 75°C for 10 min, indicating that the spores have excellent resistance to heat stress. The optimal C sources for *A. niger* growth were sucrose and fructose, which corresponded to a marked increase in fructose and glucose content in fruit during maturity (Xu et al., 2021). Compared with a single organic or inorganic N source, peptone and beef paste were optimal N sources, indicating that the nutritious compound N source was more suitable for *A. niger* growth. Coincidentally, a large amount of protein accumulated in the fruit of Shengzhou nane during maturity (Mo et al., 2017), which could provide the best N source for pathogen growth. Moreover, the isolates infected several local fruits, indicating that they had a broad host range. Therefore, it was suggested that Shengzhou nane fruit should be kept away from those fruits that are susceptible to *A. niger* during the storage and marketing process.

In Shenzhou nane production, there is an urgent need for new residue-free and safer strategies to replace conventional fungicides for the control of postharvest pathogens. This study focused on evaluating new alternatives (SBC and NT) for *A. niger* rot control in Shenzhou nane fruit, addressing both laboratory and commercial conditions. The present results indicated that 6.0 g/L or higher SBC distinctly inhibited spore germination and the colonial expansion of *A. niger*. Similarly, SBC also showed a good ability to inhibit germ tube elongation, mycelial expansion, and hypha production of *Penicillium expansum*, thus affecting the survival of microbial cells (Lai et al., 2015). Castro-Ríos et al. (2021) reported that 3.0 g/L SBC presented a significant reduction in *A. niger* growth 72 h after inoculation. Some researchers speculated that pH may be involved in the inhibitory activity of SBC since SBC could change the pH of the growth environment of microbes (Lyousfi et al., 2022). Lai et al. (2015) found that pH could induce different expressions of some genes, resulting in the inhibition of the growth of *P. expansum*. Moreover, previous studies showed that bicarbonate (HCO_3_^−^) also could dissipate the transmembrane pH gradient and dissipate *∆ψm* and ∆pH, indicating that it was a selective dissipater of the pH gradient of the proton motive force across the cytoplasmic membrane of bacteria (Farha et al., 2017). Additionally, SBC can inactivate fungal extracellular enzymes since they can act directly on cell membranes and lead to an alteration of cellular physiology Castro-Ríos et al., 2021. In addition, bicarbonate salts could reduce the intense pressure of fungal cells by increasing osmotic stress and causing the collapse and contraction of hyphae and spores (Alvindia, 2013).

Moreover, NT, a biofungicide, has been demonstrated to be effective against fungal pathogens regardless of their fungicide resistance phenotypes (Haack et al., 2018; Saito et al., 2020). It has been widely accepted that NT mainly inhibits fungal growth *via* specific binding to ergosterol, which is a necessary component of fungal membranes (Aparicio et al., 2016). In this study, 5.0 mg/L or higher NT distinctly inhibited the colonial expansion of *A. niger*, and complete inhibition of colony growth occurred at 25.0 mg/L. Moreover, 2.5 mg/L NT inhibited spore germination, indicating that spore germination was more sensitive to NT. This finding coincides with the results that a lower concentration of NT decreased the spore germination of *Alternaria* spp. and *B. cinerea* (Saito et al., 2020; Wang et al., 2021). Similarly, NT application has been reported to protect grapes from fungal rot during storage (Chang et al., 2021) and showed high antifungal efficacy on *P. expansum*, *Sclerotinia sclerotiorum*, and *Geotrichum citri-aurantii* (He et al., 2019; Seyedmohammadreza et al., 2020; Fernández et al., 2022). The elucidated mechanism of action of NT involves damage to the plasma membrane (Te Welscher et al., 2008; He et al., 2019), disturbance of nutrient transportation (Te Welscher et al., 2012), and mitochondrial dysfunction (Awasthi and Mitra, 2018). Since NT has a different mode of action from other chemical fungicides, it can be an alternative tool for controlling fungal rot in packing houses, particularly those caused by fungicide-resistant strains.

Furthermore, antimicrobial agents with synergistic effects showed more antimicrobial activities in a lower concentration than a single compound, which reduced its application cost and was more resource friendly (Kong et al., 2019). In this study, complete inhibition of colony growth occurred at 12.0 g/L SBC or 25 mg/L NT. D’Aquino et al. (2022) suggested that rinsing in a higher concentration of SBC solution could be phytotoxic to peel tissues. Therefore, the SBC concentration needs to decrease to a level that may be harmless to fruit tissues while retaining antibacterial activity by combined application. Therefore, a combination of 4.0 g/L SBC + 5.0 mg/L NT was assayed, and the results showed that SBC + NT had excellent efficacy in inhibiting spore germination and colony expansion of *A. niger* in the PDA medium. Rather than acting independently of SBC or NT, it is more likely that the different eco-friendly methods worked together synergistically.

The cell membrane plays an important role in maintaining a homeostatic environment, exchanging materials, and transferring energy and information in the cell to keep the cell healthy. The destroyed cell membrane may lead to the efflux of cellular contents, affect the membrane structure, and result in mycelial growth, eventually leading to cell death. In this study, the results of PI staining confirmed that SBC and NT could change the membrane integrity of *A. niger*. Moreover, ergosterol is an essential component of the fungal cell membrane in fungi. Ergosterol content can affect the permeability and fluidity of the membrane, which is of considerable significance for cell survival. The PI staining results, coupled with the reduction in ergosterol content in this study, indicated that the cell membrane was an important target of SBC and NT. A similar result was found by He et al. (2019), who pointed out that NT may affect membrane function by reducing ergosterol synthesis because ergosterols are important in maintaining cell function in the transport of cellular material and physiology. Reductions in total lipid and ergosterol levels usually reflect irreversible damage to cell membranes (Niu et al., 2022). However, Te Welscher et al. (2010) suggested that the antifungal mechanism of NT was that it could combine with ergosterol on the cell membrane of fungi to change the permeability of the cell membrane. Kong et al. (2021) demonstrated that SBC can directly influence the membranes of fungi by affecting the proteins that make up about 70% of most cell membranes.

In addition to acting on the cell membrane, in this study, SBC and NT treatments damaged the mitochondrial membrane integrity of *A. niger*, as shown by the results of mitochondrial red fluorescence probe staining and the fluorescence intensity of Rh-123. Mitochondria are very important organelles in eukaryotic cells and are the center of metabolism and energy conversion. Maintaining the structural integrity of mitochondria is key to maintaining cellular energy status, as well as membrane-coupled and energy-dependent processes. Therefore, in this study, coupled with the structural damage of the mitochondrial membrane, SBC and NT also caused the reduction of MMP; decreased the key enzyme activities of CS, IDH, α-KGDH, SDH, and MDH involved in the TCA cycle; and reduced the intracellular ATP content. Meanwhile, the TCA cycle is the primary metabolic and transformed pathway for generating ATP from three major nutrients: carbohydrates, lipids, and amino acids (Fernie et al., 2004). ATP content is related to the energy metabolism, release, storage, and utilization of microorganisms. The rapid reduction of key enzyme activities involved in the TCA cycle or the loss of ATP content in microorganisms leads to a lack of energy for cells for transcription and translation, cell membrane component synthesis, and so on, and eventually results in growth inhibition and cell death. The decreased MMP further proved that mitochondria were a physiological target for SBC and NT to inhibit fungal growth. The results of this study were similar to the findings in *Fusarium solani* cells with synergistic treatment by thymol and salicylic acid (Kong et al., 2021) and in *A. niger* after treatment with cinnamaldehyde in the vapor phase (Niu et al., 2022). Similarly, Tan et al. (2022) found sodium dehydroacetate interference with cell membrane metabolism and energy metabolism in the antifungal mechanism against *P. digitatum*. Cinnamon essential oil resulted in a significant decrease (*p* < 0.05) in the ATP content of *P. expansum*, suggesting that the antifungal mechanism of cinnamon essential oil against *P. expansum* might be related to the carbohydrate metabolism of fungi (Lai et al., 2021). Taken together, these findings elucidate that the damage to cell and mitochondria membrane integrity and the disruption of energy metabolism might be closely involved in the antifungal mechanism of SBC and NT against *A. niger*. However, microbial energy metabolism is a very complicated process. Further studies are necessary to investigate the TCA pathways, including the levels of TCA cycle intermediates, such as pyruvic acid, acetyl-CoA, succinate, malate, and α-ketoglutarate, and the key action sites of SBC and NT in microbial energy metabolism.

Finally, the results of this study showed the effect of SBC, NT, and their combination on postharvest disease in Shengzhou nane fruit, which was consistent with the *in vitro* test on PDA plates. In general, the combination of SBC and NT was more effective in controlling rot than individual treatments. However, it showed that the effective concentrations in the *in vivo* assay were much higher than those the *in vitro* assay. For example, 4.0 g/L SBC + 150 mg/L NT was required to control the disease caused by *A. niger* in Shengzhou nane fruit, while 4.0 g/L SBC + 2.5 mg/L NT inhibited mycelial growth completely. The effective dose of SBC and NT for the control of *A. niger* rot in inoculated fruit was higher than that derived from *in vitro* assays, possibly because the fruit provides a better environment for pathogen development and represents a hurdle for the product (He et al., 2019; Fernández et al., 2022). Our results were similar to those of Cui et al. (2021), who found that higher concentrations of magnolol were required to reduce virulence on harvested apple fruit and grapes than to suppress *in vitro B. cinerea* mycelial growth. Similarly, He et al. (2019) found that 80 mg/L NT inhibited the occurrence of gray mold disease in grapefruit, while 3.0 mg/L NT was enough to completely stunt colony growth in the PDA medium.

## 5. Conclusion

The pathogen causing fruit rot was isolated from symptomatic Shengzhou nane fruit and identified as *A. niger* by biological characteristics and molecular analysis. Optimal growth conditions for *A. niger* were 30°C, pH 5.0–6.0, and fructose and peptone as carbon and nitrogen sources. SBC, NT, and combined treatments against *A. niger* showed that the combined treatment of SBC and NT had a higher antifungal efficacy against *A. niger*. The damage to cell and mitochondrial membrane integrity and the disruption of energy metabolism might be closely involved in the antifungal mechanisms of SBC and NT. Moreover, the results of the *in vivo* experiment showed that SBC and NT reduced the rot lesion diameter and decay rate of Shengzhou nane fruit. In conclusion, the combination treatment of SBC and NT could be an alternative to synthetic fungicides for controlling postharvest Shengzhou nane decay caused by *A. niger*.

## Data availability statement

The original contributions presented in this study are included in the article material; further inquiries can be directed to the corresponding authors.

## Author contributions

T-RG performed the experiments and prepared the original draft. QZ and GY performed the experiments. S-SY and Z-YC contributed to the methodology. S-YX contributed to the resources. HW contributed to the review and editing. Y-WM contributed to the supervision, review, and editing. All authors contributed to the article and approved the submitted version.

## Funding

This study was supported by the basic public welfare research projects of Zhejiang Province, China (Nos. LGN20C150004 and LGN22C150002).

## Conflict of interest

The authors declare that the research was conducted in the absence of any commercial or financial relationships that could be construed as a potential conflict of interest.

## Publisher’s note

All claims expressed in this article are solely those of the authors and do not necessarily represent those of their affiliated organizations, or those of the publisher, the editors and the reviewers. Any product that may be evaluated in this article, or claim that may be made by its manufacturer, is not guaranteed or endorsed by the publisher.

## Footnotes

^1^ http://www.ncbi.nlm.nih.gov
