# Corrigendum: Isolation, identification, biological characteristics, and antifungal efficacy of sodium bicarbonate combined with natamycin on *Aspergillus niger* from Shengzhou nane (*Prunus salicina* var. taoxingli) fruit

**DOI:** 10.3389/fmicb.2023.1172964

**Published:** 2023-03-28

**Authors:** Tian-Rong Guo, Qing Zeng, Guo Yang, Si-Si Ye, Zi-Yi Chen, Shi-Ying Xie, Hai Wang, Yi-Wei Mo

**Affiliations:** College of Life Science, Shaoxing University, Shaoxing, China

**Keywords:** *Prunus salicina* var. taoxingli, *Aspergillus niger*, sodium bicarbonate, natamycin, antifungal mechanism

In the published article, there was an error in Equation 1. Instead of “GI = 100 ^*^ (*G* × *g*) /*G*”, it should be “GI = 100 ^*^ (*G* – *g*)/*G*”.

The authors apologize for this error and state that this does not change the scientific conclusions of the article in any way. The original article has been updated.

